# Emotional Instability Relates to Ventral Striatum Activity During Reward Anticipation in Females

**DOI:** 10.3389/fnbeh.2020.00076

**Published:** 2020-05-29

**Authors:** Frida Bayard, Christoph Abé, Nathalie Wrobel, Martin Ingvar, Eva Henje, Predrag Petrovic

**Affiliations:** ^1^Department of Clinical Neuroscience, Karolinska Institutet, Stockholm, Sweden; ^2^Department of Neuroradiology, Karolinska University Hospital, Stockholm, Sweden; ^3^Department of Clinical Science, Umeå University, Umeå, Sweden

**Keywords:** emotional instability, emotional dysregulation, ADHD, reward anticipation, functional MRI, ventral striatum

## Abstract

Both non-emotional symptoms, such as inattention, and symptoms of emotional instability (EI) are partially co-varying and normally distributed in the general population. Attention Deficit Hyperactivity Disorder (ADHD), which is associated with both inattention and emotional instability, has been related to lower reward anticipation activation in the ventral striatum. However, it is not known whether non-emotional dysregulation, such as inattention, or EI—or both—are associated with this effect. We hypothesized that altered reward processing relates specifically to EI. To test this, 29 healthy participants were recruited to this functional MRI study (*n* = 15 females). Reward processing was studied using a modified version of the Monetary Incentive Delay (MID) task. Brown Attention-Deficit Disorder Scales questionnaire was used to assess EI and inattention symptoms on a trait level. We observed less ventral striatal activation during reward anticipation related to the EI trait in females, also when controlling for the inattention trait, but not in the whole sample or males only. Our study suggests the existence of sex differences in the relationship between reward processing and EI/inattention traits.

## Introduction

Non-emotional symptoms such as inattention, impulsivity, and hyperactivity typically define Attention Deficit Hyperactivity Disorder (ADHD) according to standardized diagnostic criteria (DSM-5, 2013). ADHD affects a substantial proportion of people worldwide—5–7% of children (Spencer et al., 2007) and 2.5% of adults (Simon et al., 2009). However, in clinical practice, emotion dysregulation, including emotional instability (EI), has been reported in a subpopulation of ADHD patients (Skirrow and Asherson, 2013; Shaw et al., 2014; Moukhtarian et al., 2018). It is still in dispute whether this is part of the core ADHD disorder (Sjöwall et al., 2013; Shaw et al., 2014), or rather reflects psychiatric comorbidity, such as conduct disorder (CD), emotional instability personality disorder (EIP), anxiety disorders or major depressive disorder (MDD; Rubia, 2011; Shaw et al., 2014; Katzman et al., 2017).

The *cognitive core capacity theory* proposes a way to unify non-emotional aspects of ADHD with emotional instability (EI), i.e., rapidly shifting and intense emotional responses rather than a prolonged lowered mood (Petrovic and Castellanos, 2016). The theory also suggests that ADHD and EI related symptoms—or *traits—*are normally distributed across the general population and depend on the underlying regulatory capacities for non-emotional and emotional processes. When these traits result in the functional loss they are regarded as clinically relevant symptoms constituting a psychiatric diagnosis. Thus, to fully describe the mechanism underlying ADHD and EI disorders, the full spectrum ranging from subclinical states to the clinical disorders must be studied. This reasoning is in line with the concept of Research Domain Criteria (RDoC; Insel et al., 2010; Cuthbert and Insel, 2013), where different aspects of disorders are broken down into components that may be investigated separately on a dimensional level, and then tied together for a more consistent and structured understanding of the underlying mechanisms. It has been suggested that non-emotional ADHD symptoms and EI have both common (domain-general) and distinct (domain-specific) underlying neural mechanisms, mirrored in partially overlapping but also unique neuronal networks (Rubia, 2011; Petrovic and Castellanos, 2016). Apart from emotional top-down regulation, it has been suggested that reward processing is altered in patients with ADHD and EI (Castellanos et al., 2006; Scheres et al., 2007; Thorell, 2007; Strohle et al., 2008; Stark et al., 2011; Carmona et al., 2012; Costa Dias et al., 2013; Edel et al., 2013; Furukawa et al., 2014; White et al., 2014; Kappel et al., 2015; Yu et al., 2015; Bayard et al., 2018). Non-emotional ADHD symptoms and EI, as well as reward processing, have often been investigated in clinical populations without taking the dimensionality of symptoms in the normal population into account. The RDoC approach suggests that similar alterations could be coupled to non-emotional and EI traits also at a subclinical level. Additionally, studies including patients often include various confounds such as secondary effects of medication and co-morbidities as well as a larger effect of illicit drug use. This further highlights the importance of complementing the existing research with a dimensional approach where these confounds are not present.

Prefrontal and anterior cingulate cortex (ACC) regions, as well as the insular cortex, are involved in the representation of intrinsic reward value and subjective feeling states elicited by receiving a reward (O’Doherty et al., 2001, 2006; Craig, 2002, 2003; Kringelbach and Rolls, 2004; Kable and Glimcher, 2007; Petrovic et al., 2008; Naqvi and Bechara, 2009; Dillon et al., 2010; Namkung et al., 2017; Oldham et al., 2018). Dopaminergic circuits and the ventral striatum (VS) are essential for reward learning by mediating the reward error signal (Schultz et al., 1997; Pessiglione et al., 2006; Daniel and Pollmann, 2014; Schultz, 2016) and for motivation and salience (Berridge, 2018; Oldham et al., 2018). Functional imaging studies of reward anticipation, as well as reward receipt, involve VS activation in humans (Oldham et al., 2018).

Reduced activation of VS during reward anticipation has been shown repeatedly in ADHD patients, while activation during reward outcome has been less extensively investigated with varying results (Scheres et al., 2007; Strohle et al., 2008; Stark et al., 2011; Carmona et al., 2012; Edel et al., 2013; Furukawa et al., 2014; Kappel et al., 2015). Patients with ADHD show a relatively high preference for smaller immediate rewards over larger delayed rewards (Castellanos et al., 2006; Thorell, 2007; Yu et al., 2015)—a behavior that has been associated with altered connectivity between the nucleus accumbens (NAcc) and prefrontal regions in children with ADHD (Costa Dias et al., 2013). Since ADHD is highly correlated with EI traits, it remains unclear whether the aberrant activation of VS is related to EI rather than to typical non-emotional ADHD symptoms. This question pertains both to ADHD patients and to ADHD traits. In line with this reasoning, the tendency to discount the value of a delayed reward is associated with both ADHD traits and CD traits (characterized by EI) respectively in a large community sample (Bayard et al., 2018) and similar results have been verified in CD and ADHD patients without psychiatric co-morbidity (White et al., 2014).

The overall aim of the present study was to investigate the relationship between EI and non-emotional traits in reward anticipation in a non-clinical sample. Inattention was chosen to best represent the non-emotional dimension, as previously done (Petrovic et al., 2016). We hypothesized that the EI trait would be uniquely linked to a lower reward anticipation signal in the VS also when controlling for the inattention trait. We also hypothesized that ACC and insular related activity during reward outcome would be associated with the EI trait, rather than inattention. We used a modified version of the Monetary Incentive Delay (MID) task (Knutson et al., 2001a,b), which has been specifically designed to study neural correlates of reward anticipation and outcome with fMRI. Since sex differences have been reported both about ADHD symptom profiles and reward processing (Rucklidge, 2010; Bobzean et al., 2014; Davies, 2014; Mowlem et al., 2018; Becker and Chartoff, 2019), we also explored sex differences in reward processing concerning EI and inattention traits.

## Materials and Methods

### Participants

Twenty-nine healthy participants were included in the present study (see Supplementary Material for details of recruitment). All subjects gave written and oral consent for participation in the study, which was conducted according to the Helsinki Declaration and approved by the regional ethics committee in Stockholm (application number 2015/127-31/1, amendment number 2016/1711-32). Each participant received a total of 840 SEK (≈84 Euro) for participation in the study. This sum was equivalent to the total win in the MID task (see below).

### Assessment

Inattention and EI traits were assessed with the Brown Attention-Deficit Disorder Scales (Brown-ADD) self-report questionnaire (Brown, 1996). The questionnaire contains 40 items, divided into five subscales assessing different aspects of ADD symptoms—*Activation* (Organizing, Prioritizing and Activating to Work), *Attention*, herein referred to as *Inattention* (Focusing, Sustaining and Shifting Attention to Tasks), *Effort* (Regulating Alertness, Sustaining Effort, and Processing Speed), *Affect*, herein referred to as *Emotion Instability* (Managing Frustration and Modulating Emotions), *Memory* (Utilizing Working Memory and Accessing Recall). A total score combining all five subscales can be obtained. Answers range from 0 (“never”) to 3 (“almost daily”). For this study, we were interested in the *Inattention* and the *Emotion Instability* subscales to represent non-emotional ADHD trait and EI trait, respectively, as done previously (Petrovic et al., 2016). The maximum score for *Inattention* is 27, the maximum score for *Emotion* *Instability* is 21. Higher subscale scores indicate more difficulties in that particular domain.

### Experimental Stimuli and Task

**Figure 1:**
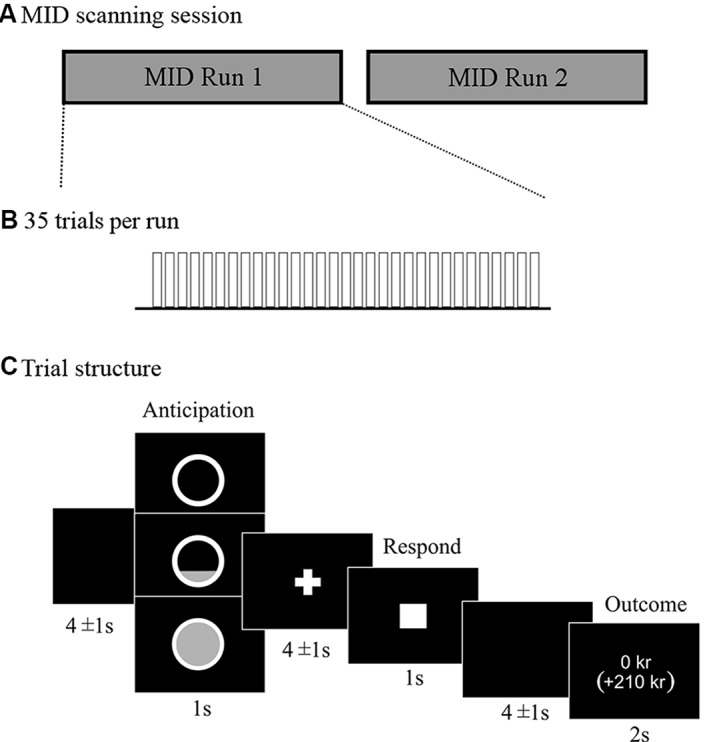
Description of the monetary incentive delay (MID) scanning session **(A)** and trial structure of the modified version of the MID task (**B,C**). Presentation software (Neurobehavioral Systems) was used to present the task in the scanner. The whole MID task required ≈35 min of scanning. Each of the two runs consisted of 35 trials of ≈16 s each, which resulted in 9.3 min per run. This led to an approximate scanning time of 10 min per run including instructions at the beginning of each run and repetition of trials due to possible mistakes of the participant. There was a total of 70 trials. The outcome was not dynamically adjusted but fixed to yield the same number of wins for each participant with a success rate of 50% for each reward level. Participants were told that different trials would vary in difficulty, and they were instructed to always respond as fast as they could to win as much as possible, even if some trials would require an impossibly fast response and be highly unlikely to result in a win. Participants were explicitly instructed to only press the button once for each trial. There were 14 *Baseline* trials where participants anticipated no win, and could not win anything, but were encouraged to press the button nevertheless in the same way as in the reward trials. There were 28 trials with the opportunity to win 10 SEK (subsequently referred to as *Low win*, equivalent to ≈1 Euro), and 28 trials with the opportunity to win 50 SEK (subsequently referred to as *High win*, equivalent to ≈5 Euro). As the actual winning rate was fixed, 50% of the trials resulted in *“Failed” win* and consequently no reward outcome. The uncertainty of the outcome was important to keep participants motivated to perform. The outcome phase included a presentation of the winning amount in the current trial, as well as the total accumulated sum below in parentheses.

We used a modified version of the MID task, originally developed by Knutson and colleagues (Knutson et al., 2001a,b). Two pseudo-randomized presentation orders were used to avoid possible order effects. The details of the modified version of the MID task are presented in Figure 1.

### MRI Data Acquisition

All participants practiced a test version of the MID task of about 2.5 min on a computer and were subsequently placed inside a 3T GE scanner (Discovery MR750; GE, Fairfield, CT). Anatomical and fMRI scans were acquired using an eight-channel head coil. In addition to the fMRI scan, a T1-weighted scan was performed and later used for co-registration of functional MRI data for each participant.

For the functional scans, 45 slices were acquired in an interleaved order using a gradient-echo EPI sequence (flip angle = 70°, echo time = 30 ms, repetition time = 2,200 ms) with a field of view of 23.0 cm, matrix size of 76 × 76, slice thickness of 3 mm, slice spacing 0.3 mm. The T1-weighted images were acquired using a 3D-BRAVO sequence (TR = 6.40 ms, TE = 2.81 ms, FOV = 24.0 cm, flip angle = 12°, inversion time 450 ms, acquisition matrix 240 × 240 × 180, with isotropic voxel size 1 × 1 × 1 mm^3^. The B0 Fieldmap sequence was used to acquire 45 slices in an interleaved manner using a gradient-echo EPI sequence (TR = 755.6 ms, TE = 7.0 ms, flip angle = 45°, FOV = 22.0 cm, acquisition matrix 64 × 64, slice thickness 3.0 mm, slice spacing 0.3 mm).

### MRI Data Pre-processing

The collected data was arranged to follow the Brain Imaging Data Standard (BIDS; Gorgolewski et al., 2016). The re-organized data was run through the fMRIPrep pipeline, version 1.0.11 (Esteban et al., 2019). Processing steps included skull-stripping, brain tissue segmentation, normalization to MNI space, brain-mask extraction of BOLD images, motion-correction, segmentation, and co-registration of BOLD images to T1. The B0 field map was included to account for susceptibility distortion fields. For details on fMRIPrep’s processing steps, see Supplementary Material Section 2.2.1. The output from fMRIPrep was further smoothed in SPM12 (Statistical parametric mapping, The Welcome Department of Imaging Neuroscience, Institute of Neurology, University College London) running on Matlab 14 (MATLAB 2014, The MathWorks Inc., MA, USA), using a Gaussian smoothing kernel of FWHM = 6 mm. An automated quality control procedure included in the fMRIPrep pipeline enabled the quality check of all participants’ scans, focusing on framewise displacement outliers.

### Data Analysis

Methods to confirm that our version of the MID-task-evoked similar behavioral and fMRI responses as previous MID studies are described in detail in Supplementary Materials.

### Behavioral Data

To test our main hypothesis that altered reward processing is related to EI rather than inattention trait on a behavioral level, we correlated *mean reaction time (RT*; *Baseline* and *Win* trials), *reaction time variability* (*RTV*; defined as the standard deviation of *RT*) and *response time speeding* (*RTS*; *Baseline* *RT—*mean *Win* *RT*) with B-ADD scores of *Inattention* and *Emotion Instability*. For these analyses, we ran multiple regressions using the “lm” function in R version 3.1.3 (R Core Team, 2015). *RT*, *RTV*, and *RTS* were dependent variables, and *Inattention* and *Emotion Instability* were independent variables in the models. We further controlled for age and sex by adding those as independent variables. *RTS* represents the relative difference between a non-incentive condition and the high and low incentive conditions for each participant and a similar approach has been used previously to quantify differences in behavior in the MID task (Veroude et al., 2016; van Hulst et al., 2017). When response times were longer than 1 s the target was no longer presented on the screen and such trials did not lead to a reward. Since too slow responses during low-incentive conditions could reflect low motivation, removing these trials could result in incorrect representations of average *RT* and *RTV* and they were therefore included in the analyses. Participants were explicitly instructed to press the response button only once for each trial, and multiple response trials were excluded since they represented the inability to follow these instructions.

### fMRI Data

The onset times of events of interest in the MID task were those of anticipation of reward and reward outcomes. The anticipation phase was modeled as a stick function and convoluted with the canonical hemodynamic response function (HRF) to the onset of the reward anticipation cue of either *Baseline*, *Low win*, or *High win*. In line with the behavioral analysis, trials with an *RT* of >1 s were included in the anticipation phase analysis, while multiple response trials or trials with omitted responses were excluded. Similarly, successful outcome conditions of *Low win* and *High win* were modeled separately as stick functions, convolved with the HRF. Even if the *Baseline* outcome and the outcome of *“Failed” win* trials all resulted in no-win, these conditions were modeled separately since they were thought to represent different underlying neural processes. Button press and motion parameters were included in the model as regressors of no interest.

Main first-level contrasts were defined by combining the two win conditions (*Low win* and *High win*) and contrasting those against *Baseline* (*Win* vs. *Baseline*) both for the reward anticipation phase and the reward outcome phase. The two win conditions were combined since our hypothesis only concerned reward in general and not a specific level of reward. The *“Failed” win* trials resulting in no-win outcome were not considered in the *a priori* hypotheses, but brain activation associated with “failed” reward outcome (*“Failed” win vs Baseline* contrast) was explored in follow-up analyses and reported in Supplementary Materials for completeness.

We used an ROI approach for our main analyses. The pre-defined ROI for the anticipation of reward was based on peak activations in left and right VS reported in a recent meta-analysis on MID task brain activation (Oldham et al., 2018). We created a bilateral ROI around the reported peak activations (spherical ROIs, radius = 6 mm, Supplementary Figure S1A). For the reward outcome phase, we based the ROIs on peak activations located in rostral ACC (rACC) and bilateral insula (spherical ROIs, radius = 10 mm, the left and right insula were combined as one ROI) reported in a study (Dillon et al., 2010) that applied similar jittering and baseline strategies for the reward outcome phase as in the present study (Supplementary Figure S1B).

To test our *a priori* hypotheses, we ran t-tests on the second level including B-ADD *Inattention* and *Emotion Instability* scores as regressors of interest within the pre-defined ROIs. Small volume correction was applied within these regions, and clusters were considered significant if the FWE corrected peak *p*-value was <0.05. As a next step, we tested whether correcting for age and sex affected brain activation. Since reward processing differs between sexes (Bobzean et al., 2014; Becker and Chartoff, 2019) and the symptomatology profile differs between female and male ADHD patients (Rucklidge, 2010; Trent and Davies, 2012; Davies, 2014) we explored the effects of sex-by-*Emotion Instability* and sex-by-*Inattention* interaction on brain activity. Finally, we ran a whole-brain analysis to explore whether activation of additional regions showed correlations with B-ADD *Emotion Instability* or *Inattention* scores.

We complemented the small volume correction ROI approach by analyzing extracted mean parameter estimates from the pre-defined ROIs. This analysis was used to compare activation related to the different reward levels, as well as for follow-up analysis of the reward anticipation signal changes in VS. This commonly used approach in fMRI analyses provides information on more general increases or decreases in signal over a larger brain region, enabling noise reduction especially in relatively small, functionally distinct regions (Poldrack, 2007; Poldrack et al., 2011).

## Results

### Description of the Sample

**Figure 2:**
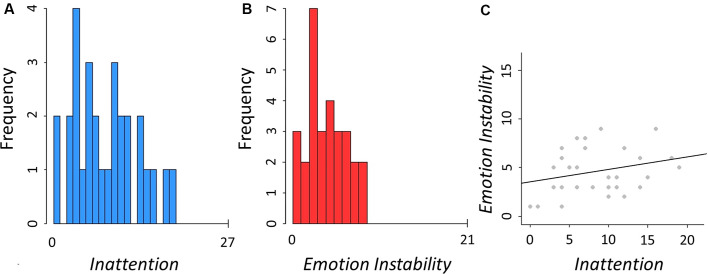
Descriptives of the assessed traits. **(A)** Distribution of B-ADD *Inattention*, mean = 8.59 (SD = 5.03, min = 0, max = 19). **(B)** Distribution of B-ADD *Emotion Instability*, mean = 4.59 (SD = 2.37, min = 1, max = 9). **(C)** Correlation between *Inattention* and *Emotion Instability* (*r* = 0.27, *p* = 0.16).

Twenty-nine participants were included in the analyses (15 females), mean age 28.94 (SD = 6.47, min = 18.70, max = 46.50). Distribution of B-ADD subscales *Inattention* and *Emotion Instability* and the correlation between these two dimensions in each participant (*r* = 0.27, *p* = 0.16) are shown in Figure 2. There was no significant statistical difference between sexes in these measures (*Inattention*: *t*_(27)_ = 0.60, *p* = 0.55, *Emotion Instability*: *t*_(27)_ = 0.97, *p* = 0.34). Additional descriptive characteristics of the sample can be found in Supplementary Table S1.

### Behavioral Results

**Figure 3:**
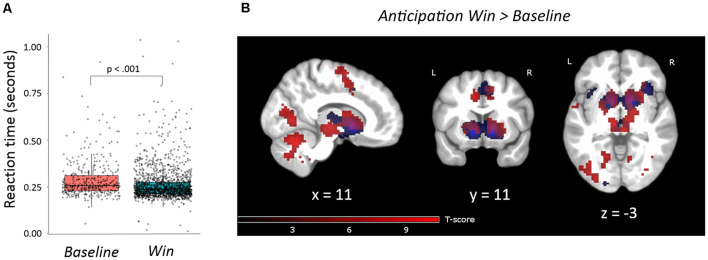
**(A)** Reaction times for win trials were statistically shorter than for baseline trials (beta = −0.03, *t*_(1936)_ = −5.72, *p* < 0.001). The linear mixed-effects model was statistically significant (*F* = 32.70, *p* < 0.001). **(B)** Task activations of contrast anticipation *Win vs. Baseline*. FWE, *p* = 0.05, clusters >20 voxels. Activations are overlaid on an average T1 image based on our 29 participants. Task activations (red color) in this study overlap with activations reported by Oldham and colleagues (blue color; Oldham et al., 2018). For the exact location of activations, see Supplementary Table S3.

To confirm that our version of the MID task evoked similar behavioral responses as previous versions we ran a linear mixed effects model (described in Supplementary Materials). This analysis showed a significant effect of *Win* vs. *Baseline* on *RT* (M_*RTWin*_ = 0.26 s (s), SD = 0.05); M_*RTBaseline*_ = 0.29 s, SD = 0.10); *t*_(1,936)_ = −5.72, *p* < 0.001; Figure 3A). Further detailed results of the different reward levels are presented in Supplementary Materials, Supplementary Figure S2 and Supplementary Table S2. The main behavioral analysis showed no significant correlations between the traits of interest (B-ADD *Inattention* or *Emotion Instability*) and *RT* or *RTV* and adjusting for age and sex did not change the results. There was no main effect of sex or a sex-by-*Emotion Instability*/*Inattention* interaction on *RT* or *RTV*.

A trend level moderate correlation was observed between *RTS* and *Emotion Instability* (*r* = 0.32, *p* = 0.09), which was still present when controlling for *Inattention* (standardized beta-weight = 0.35, *p* = 0.08). There was no significant correlation between *Inattention* and *RTS* and no main effect of sex or sex by *Emotion Instability*/*Inattention* interaction.

### fMRI Results

#### Main Activations

**Figure 4:**
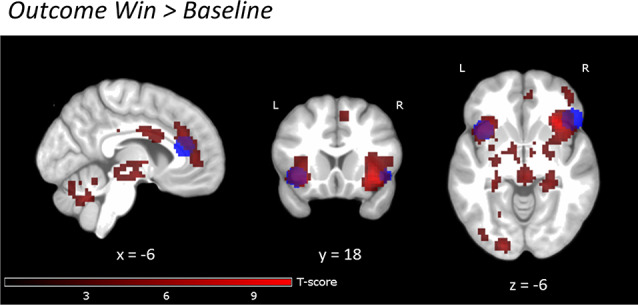
Reward outcome *Win* vs. *Baseline*. Uncorrected, *p* < 0.001, clusters >20 voxels. Activations are overlaid on an average T1 image based on our 29 participants. Task activations (red color) overlap with activations in the study by Bayard et al., 2018 (blue color) in bilateral insula and rACC. For the exact location of activations, see Supplementary Table S4.

During reward anticipation, the whole-brain analysis showed nine large significant main activation clusters (FWE-corrected) for the contrast *Win* vs. *Baseline* expanding over bilateral VS and adjacent regions, thalamus, ACC, right anterior insula, pre-frontal, motor, parietal, occipital, cerebellar and brain stem regions (Figure 3B and Supplementary Table S3). In the reward outcome condition (excluding *“Failed” win* trials, see “Materials and Methods” section) the whole-brain analysis showed significant activations (FWE-corrected) for *Win* vs. *Baseline* in a network including the bilateral anterior insula and rACC extending into dorsal ACC (dACC; Figure 4, Supplementary Table S4). Results were similar for *“Failed” win* outcomes (Supplementary Figure S6, Supplementary Table S5, see Supplementary Materials for more detail), though medial activations were slightly more dorsal than for successful reward outcomes. Further detailed results of the different reward levels are presented in Supplementary Materials and Supplementary Figures S3, S4 and S5.

#### Relation Between Reward Processing Brain Activation and *Emotion Instability*/*Inattention* Traits

Reward anticipation: we did not find any significant correlations between the traits of interest (*Emotion Instability* or *Inattention*) and activations in the contrast anticipation *Win* vs. *Baseline* within the VS ROI. There were no significant correlations of reward anticipation activation and age within the predefined ROI.

Reward outcome: there were no correlations of *Emotion Instability* or *Inattention* within the pre-defined ROIs in rACC and bilateral insula for successful reward outcomes (*Win* vs. *Baseline*). There were no significant correlations of reward outcome activation and age within our pre-defined ROIs.

#### Sex Differences in Correlations Between Brain Activation and *Emotion Instability*/*Inattention*

We found a significant effect of sex (controlling for *Emotion Instability* and *Inattention*) on activation during reward anticipation in the left VS (Supplementary Figure S7). Analysis of the mean parameter estimates, i.e., the average signal change, from the pre-defined bilateral VS ROIs during reward anticipation revealed a trend significant effect of sex (controlling for *Emotion Instability* and *Inattention*, standardized beta-weight = 0.43, *p* = 0.053) as well as an additional interaction effect of sex-by-*Emotion Instability* (controlling for sex, *Emotion Instability*, and *Inattention*, *p* = 0.01) on average signal change in VS during reward anticipation. See also Supplementary Figure S8 for visualization of the corresponding small volume corrected voxel-wise analysis within the bilateral VS ROI.

There were no effects of sex, sex-by-*Emotion Instability* interaction, or sex-by-*Inattention* interaction on reward outcome activation within our pre-defined ROIs.

**Figure 5:**
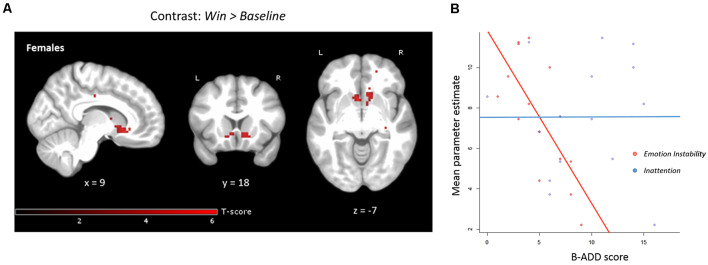
**(A)** Negative correlation with *Emotion Instability*, controlling for *Inattention* in anticipation *Win* vs. *Baseline* for females only (*n* = 15). Both the right and left VS clusters were significant when applying small volume correction (within the bilateral pre-defined VS ROI). Note that no mask is applied in the above figure for clarity, uncorrected level, *p* < 0.001. **(B)** Mean parameter estimates extracted from the pre-defined bilateral VS ROI during reward anticipation correlated negatively with *Emotion Instability* (*r* = −0.71, *p* < 0.01, when controlling for *Inattention*: standardized beta-weight = 0.73, *p* < 0.01). There was no significant correlation of mean parameter estimates extracted from bilateral VS and *Inattention* (*r* = 0.006, *p* = 0.98). The maximum B-ADD score for *Emotion Instability* was 21 (maximum in the current sample: 9) and for *Inattention* 27 (maximum in the current sample: 16).

In females, we observed statistically significant negative correlations between *Emotion Instability* and activation bilaterally within the pre-defined VS ROI for the anticipation of *Win* vs. *Baseline*, applying small volume correction (Table 1). The results were similar when controlling for *Inattention* (Figure 5, Table 1) or age. There were no significant correlations of *Inattention* and brain activation during anticipation of reward when controlling for *Emotion Instability* in the female subsample.

**Table 1:** Correlations of *Emotion Instability* and BOLD signal within the bilateral VS during reward anticipation in females.

Localization	MNI			Peak z-score	Cluster size (voxels)	Peak *p*-value, FWE corrected
	*X*	*Y*	*Z*			
*Emotion Instability*—BOLD signal correlation
Right VS	10	13	−2	3.57	21	0.011
Left VS	−5	13	−5	4.03	20	0.002
*Emotion Instability*—BOLD signal correlation, controlling for *Inattention*
Right VS	10	13	−2	3.43	24	0.018
Left VS	−5	13	−5	3.97	24	0.002

*Cluster size at uncorrected level *p* < 0.05. Small volume correction applied. Voxel size 3 × 3 × 3 mm^3^. VS, ventral striatum*.

In males, there were no significant correlations between *Emotion Instability*/*Inattention* and brain activation during anticipation of reward.

## Discussion

The present study found partial support for the hypothesis that emotional instability (EI) is related to decreased activity in the ventral striatum (VS) during reward anticipation. There was an interaction effect of sex by *Emotion Instability* score and in female subjects, VS activity related negatively to Brown Attention-Deficit Disorder Scales (B-ADD)* Emotion Instability* score, even when adjusting for non-emotional B-ADD *Inattention* score. However, this relation could not be observed in the sample as a whole or males. Activation in the rostral ACC (rACC) and bilateral insula during reward outcome did not correlate with *Emotion Instability*, and there was no indication of a sex-by-*Inattention*/*Emotion Instability* interaction for the outcome phase.

The here applied MID task produced similar main activations as in previous studies for the reward anticipation phase, including VS activation (Oldham et al., 2018). However, in contrast to previous studies, we did not observe VS activation during the outcome phase. One reason for the diverse results may be that most studies have not jittered between all phases of the MID task, which makes it difficult to separate brain activations from the different task events. Thus, VS activation during the outcome phase observed in previous studies might be confounded by VS activation associated with anticipation. The present version of the task overcomes this issue and indicates that VS activation is related to anticipation rather than a rewarding outcome. Also, we separated the “failed” reward outcome from a successful reward outcome and found that both conditions elicit overlapping activations in ACC and insula. This implies a more general involvement of these regions in different types of emotional and feeling states.

The main finding in the present study was the negative correlation between *Emotion Instability* scores and VS activation during reward anticipation in females, also when controlling for *Inattention* scores. Previous functional imaging studies have shown that both clinical ADHD and high ADHD traits are related to abnormally low activation in VS during reward anticipation (Scheres et al., 2007; Strohle et al., 2008; Stark et al., 2011; Carmona et al., 2012; Edel et al., 2013; Furukawa et al., 2014; Kappel et al., 2015). Those studies have rarely considered that emotional regulation capacity could potentially influence the results. Our findings are in line with the cognitive core capacity theory, which suggests that mechanisms underlying non-emotional ADHD traits, such as inattention, and EI are partially shared and partially domain-specific (Petrovic and Castellanos, 2016). The current study supports the suggestion that emotional and non-emotional symptoms should be considered separately. Also, our results may have clinical implications, since the core ADHD diagnostic criteria do not consider emotional aspects of psychopathology which could potentially identify ADHD subtypes requiring different treatments. Also, our dimensional approach to parse different traits of ADHD in a population sample and investigate their neural correlates is supported by the Research Domain Criteria (RDoC) concept (Insel et al., 2010; Cuthbert and Insel, 2013) as a way to better understand psychiatric disorders.

Our results suggest that there is an association between the capacity of emotion regulation (mirrored in the degree of EI) and low-level reward processing (mirrored in VS hypoactivation during reward anticipation) and that this relationship is independent of non-emotional regulation capacity (mirrored in the degree of non-emotional inattention symptoms). To the best of our knowledge, it is the first time that this association has experimental support. We suggest that our observations may result from suboptimal dopaminergic signaling. The relation between emotion regulation capacity and reward anticipation processing may be mirrored in the function of different dopamine pathways projecting towards prefrontal regions and ACC (such as medial PFC, orbitofrontal cortex and rACC), and dopamine pathways projecting towards VS—involved in simple reward anticipation and reward error signaling (Hauser et al., 2016). Besides, individuals may also present with varying functions in dopamine projections primarily towards either emotional or non-emotional top-down regulatory networks (Petrovic and Castellanos, 2016). Although these interact, the functional balance between them may partially differ between individuals and subsequently affect also reward processing to a varying degree.

Finally, our results underscore the need to disentangle the underlying mechanisms of emotional and non-emotional symptomatology related to ADHD and EI about sex. This is also emphasized by previous research suggesting sex differences in reward processing (Bobzean et al., 2014; Becker and Chartoff, 2019), ADHD symptomatology (Rucklidge, 2010; Davies, 2014), and ADHD treatment efficacy (Hodgson et al., 2014; Mowlem et al., 2018).

There were several limitations to this study. Due to small subsamples in the explorative analyses, potential sex differences indicated here should be treated with caution until further replication. Further, the B-ADD self-rating scale used in this study might only partly reflect the constructs of interest. To better understand underlying neural processes, there is a need to develop tools that better quantify and successfully isolate EI and non-emotional ADHD traits.

Also, depressive symptoms are known to influence reward processing (Whitton et al., 2015), which could potentially have contributed to our findings. However, since all participants reported no previous or current depression this is unlikely. Also, fluctuations of hormonal levels over the menstrual cycle could have influenced reward processing (Sacher et al., 2013; Bobzean et al., 2014; Becker and Chartoff, 2019).

## General Conclusion and Implications

We observed that less ventral striatal activation during reward anticipation was related to subclinical emotional instability in females. The study suggests that emotional and non-emotional symptoms should be disentangled, concerning sex, in both non-clinical and clinical study groups. The use of a dimensional approach when quantifying psychopathology may be a step towards the development of more effective treatment paradigms.

## Data Availability Statement

The datasets generated for this study are available on request to the corresponding author.

## Ethics Statement

The studies involving human participants were reviewed and approved by the ethics committee in Stockholm (application number 2015/127-31/1, amendment number 2016/1711-32). The patients/participants provided their written informed consent to participate in this study.

## Author Contributions

FB and PP were mainly responsible for the conception and design of the study. FB, CA and NW acquired and analyzed the data. FB, CA, NW, MI, EH and PP all contributed to interpreting the results and writing the manuscript. All authors have approved the submitted version.

## Conflict of Interest

The authors declare that the research was conducted in the absence of any commercial or financial relationships that could be construed as a potential conflict of interest.
