# Supplementary material for: Emotional Instability Relates to Ventral Striatum Activity During Reward Anticipation in Females
Source: Front Behav Neurosci. 2020 May 29;14:76. doi: 10.3389/fnbeh.2020.00076 (PMC7274270; doi:10.3389/fnbeh.2020.00076)
Supplement: Supplementary file 1 [file Data_Sheet_1.docx]

Supplementary Material

1. **Materials and Methods**

**1.1 Participants and recruitment**

Participants were recruited from four university sites in the Stockholm area, through an online recruitment site for scientific studies, as well as through the homepage of Karolinska Institutet.

Inclusion criteria were adults of both sexes without ADHD, emotional instability disorder or any other current or previous psychiatric disorder. In order to avoid confounding factors, we also excluded potential participants with current nicotine or drug use, dyslexia, Body Mass Index <17 or >35 (which could be related to hormonal disturbances), neurological or endocrinological disease or long-term medication. Short-term analgesics and allergy medication were allowed, except on the day of participation in the study. Oral contraceptives were permitted. fMRI related exclusion criteria were left-handedness, known color blindness or previous traumatic brain injury that led to hospitalization. Severe vision problems and non-fluency in Swedish language reduced the ability to complete the fMRI-task and therefore served as exclusion criteria. Finally, standard fMRI security exclusion criteria were applied such as presence of metallic implants, devices or splinters, pacemaker, non-removable piercings and pregnancy.

Potential participants contacted the test leader directly, and a first telephone screening was performed. The screening took about 30 minutes and included information about the study, oral informed consent, questions about the general health of the participant, and a first MRI safety screening. Prospective subjects who displayed the above mentioned characteristics where invited to the test site at the MR center at Karolinska Hospital Solna. At arrival at the test site, the participant was informed about the study procedure and gave written informed consent. After a final MRI safety screening was performed, the participant completed a questionnaire, see assessment.

Twenty-nine of the 31 participants who visited the MR-center were included in the final analyses. One participant could not complete the scanning due to technical scanner problems and one participant was excluded due to previously unidentified severe self-reported gambling problems.

**1.2 MRI data acquisition**

An anatomical T2 scan was sent to the Karolinska Hospital and screened for abnormalities by a neuro-radiologist. In case aberrations would have been detected, the participant would have been contacted for a follow-up scan, following Swedish health care routine. No such findings were observed in the present study. Data was collected during the autumn of 2016 and winter 2017.

1. **Data Analysis**

**2.1 Behavioral**

To test whether the present version of the MID task could elicit similar behavioral responses as previous MID tasks, we measured reaction time (*RT)* in win trials and baseline trials. For follow-up analysis *RT* was also calculated for *High win* and *Low win* separately. We used a Linear Mixed effects model, nlme package (Pinheiro J 2019) in R version 3.1.3 (R Core Team 2015), method “REML”. Assuming that trial type is the driving factor for RT differences between trial types, and to account for individual differences in processing speed, we used a linear mixed effects model with intercept of participant as random factor and reward level as fixed effect. *RT* was the dependent variable and all RTs for all events and participants were included in the mixed effects model analysis, resulting in a total of 1966 “correct response” observations in total (29 participants responding to 70 trials each).

***2.1.1 Correlations RT measures and Emotion/Attention***

To further explore the behavioral data, we also computed the variables ***high RTS*** (*Baseline* *RT* – *High win* *RT*) and ***low RTS*** (*Baseline* *RT* – *Low win* *RT*), additionally to the main analysis of *RTS* combining both *High* *win* and *Low win* trials.

- 1. **MRI**
     1. ***fMRIprep methods***

fMRIprep’s methods as extracted directly for fMRIprep version 1.0.11 from <https://fmriprep.readthedocs.io/en/1.0.11/citing.html>, including associated references:

Results included in this manuscript come from preprocessing performed using FMRIPREP version latest [1], a Nipype [2,3] based tool. Each T1w (T1-weighted) volume was corrected for INU (intensity non-uniformity) using N4BiasFieldCorrection v2.1.0 [4] and skull-stripped using antsBrainExtraction.sh v2.1.0 (using the OASIS template). Spatial normalization to the ICBM 152 Nonlinear Asymmetrical template version 2009c [6] was performed through nonlinear registration with the antsRegistration tool of ANTs v2.1.0 [7], using brain-extracted versions of both T1w volume and template. Brain tissue segmentation of cerebrospinal fluid (CSF), white-matter (WM) and gray-matter (GM) was performed on the brain-extracted T1w using fast [16] (FSL v5.0.9).

Functional data was motion corrected using mcflirt (FSL v5.0.9 [8]). This was followed by co-registration to the corresponding T1w using boundary-based registration [15] with 9 degrees of freedom, using flirt (FSL). Motion correcting transformations, BOLD-to-T1w transformation and T1w-to-template (MNI) warp were concatenated and applied in a single step using antsApplyTransforms (ANTs v2.1.0) using Lanczos interpolation.

Physiological noise regressors were extracted applying CompCor [17]. Principal components were estimated for the two CompCor variants: temporal (tCompCor) and anatomical (aCompCor). A mask to exclude signal with cortical origin was obtained by eroding the brain mask, ensuring it only contained subcortical structures. Six tCompCor components were then calculated including only the top 5% variable voxels within that subcortical mask. For aCompCor, six components were calculated within the intersection of the subcortical mask and the union of CSF and WM masks calculated in T1w space, after their projection to the native space of each functional run. Frame-wise displacement [18] was calculated for each functional run using the implementation of Nipype.

Many internal operations of FMRIPREP use Nilearn [21], principally within the BOLD-processing workflow. For more details of the pipeline see https://fmriprep.readthedocs.io/en/latest/workflows.html.

1. FMRIPREP Available from: 10.5281/zenodo.852659.

2. Gorgolewski K, Burns CD, Madison C, Clark D, Halchenko YO, Waskom ML, Ghosh SS. Nipype: a flexible, lightweight and extensible neuroimaging data processing framework in python. Front Neuroinform. 2011 Aug 22;5(August):13. doi:10.3389/fninf.2011.00013.

3. Gorgolewski KJ, Esteban O, Ellis DG, Notter MP, Ziegler E, Johnson H, Hamalainen C, Yvernault B, Burns C, Manhães-Savio A, Jarecka D, Markiewicz CJ, Salo T, Clark D, Waskom M, Wong J, Modat M, Dewey BE, Clark MG, Dayan M, Loney F, Madison C, Gramfort A, Keshavan A, Berleant S, Pinsard B, Goncalves M, Clark D, Cipollini B, Varoquaux G, Wassermann D, Rokem A, Halchenko YO, Forbes J, Moloney B, Malone IB, Hanke M, Mordom D, Buchanan C, Pauli WM, Huntenburg JM, Horea C, Schwartz Y, Tungaraza R, Iqbal S, Kleesiek J, Sikka S, Frohlich C, Kent J, Perez-Guevara M, Watanabe A, Welch D, Cumba C, Ginsburg D, Eshaghi A, Kastman E, Bougacha S, Blair R, Acland B, Gillman A, Schaefer A, Nichols BN, Giavasis S, Erickson D, Correa C, Ghayoor A, Küttner R, Haselgrove C, Zhou D, Craddock RC, Haehn D, Lampe L, Millman J, Lai J, Renfro M, Liu S, Stadler J, Glatard T, Kahn AE, Kong X-Z, Triplett W, Park A, McDermottroe C, Hallquist M, Poldrack R, Perkins LN, Noel M, Gerhard S, Salvatore J, Mertz F, Broderick W, Inati S, Hinds O, Brett M, Durnez J, Tambini A, Rothmei S, Andberg SK, Cooper G, Marina A, Mattfeld A, Urchs S, Sharp P, Matsubara K, Geisler D, Cheung B, Floren A, Nickson T, Pannetier N, Weinstein A, Dubois M, Arias J, Tarbert C, Schlamp K, Jordan K, Liem F, Saase V, Harms R, Khanuja R, Podranski K, Flandin G, Papadopoulos Orfanos D, Schwabacher I, McNamee D, Falkiewicz M, Pellman J, Linkersdörfer J, Varada J, Pérez-García F, Davison A, Shachnev D, Ghosh S. Nipype: a flexible, lightweight and extensible neuroimaging data processing framework in Python. 2017. doi:10.5281/zenodo.581704.

4. Tustison NJ, Avants BB, Cook PA, Zheng Y, Egan A, Yushkevich PA, Gee JC. N4ITK: improved N3 bias correction. IEEE Trans Med Imaging. 2010 Jun;29(6):1310–20. doi:10.1109/TMI.2010.2046908.

5. Dale A, Fischl B, Sereno MI. Cortical Surface-Based Analysis: I. Segmentation and Surface Reconstruction. Neuroimage. 1999;9(2):179–94. doi:10.1006/nimg.1998.0395.

6. Fonov VS, Evans AC, McKinstry RC, Almli CR, Collins DL. Unbiased nonlinear average age-appropriate brain templates from birth to adulthood. NeuroImage; Amsterdam. 2009 Jul 1;47:S102. doi:10.1016/S1053-8119(09)70884-5.

7. Avants BB, Epstein CL, Grossman M, Gee JC. Symmetric diffeomorphic image registration with cross-correlation: evaluating automated labeling of elderly and neurodegenerative brain. Med Image Anal. 2008 Feb;12(1):26–41. doi:10.1016/j.media.2007.06.004.

8. Jenkinson M, Bannister P, Brady M, Smith S. Improved optimization for the robust and accurate linear registration and motion correction of brain images. Neuroimage. 2002 Oct;17(2):825–41. doi:10.1006/nimg.2002.1132.

9. Andersson JLR, Skare S, Ashburner J. How to correct susceptibility distortions in spin-echo echo-planar images: application to diffusion tensor imaging. Neuroimage. 2003 Oct;20(2):870–88. doi:10.1016/S1053-8119(03)00336-7.

10. Cox RW. AFNI: software for analysis and visualization of functional magnetic resonance neuroimages. Comput Biomed Res. 1996 Jun;29(3):162–73. doi:10.1006/cbmr.1996.0014.

11. Jenkinson M. Fast, automated, N-dimensional phase-unwrapping algorithm. Magn Reson Med. 2003 Jan;49(1):193–7. doi:10.1002/mrm.10354.

12. Huntenburg JM. Evaluating nonlinear coregistration of BOLD EPI and T1w images. Freie Universität Berlin; 2014. Available from: http://hdl.handle.net/11858/00-001M-0000-002B-1CB5-A.

13. Wang S, Peterson DJ, Gatenby JC, Li W, Grabowski TJ, Madhyastha TM. Evaluation of Field Map and Nonlinear Registration Methods for Correction of Susceptibility Artifacts in Diffusion MRI. Front Neuroinform. 2017 [cited 2017 Feb 21];11. doi:10.3389/fninf.2017.00017.

14. Treiber JM, White NS, Steed TC, Bartsch H, Holland D, Farid N, McDonald CR, Carter BS, Dale AM, Chen CC. Characterization and Correction of Geometric Distortions in 814 Diffusion Weighted Images. PLoS One. 2016 Mar 30;11(3):e0152472. doi:10.1371/journal.pone.0152472.

15. Greve DN, Fischl B. Accurate and robust brain image alignment using boundary-based registration. Neuroimage. 2009 Oct;48(1):63–72. doi:10.1016/j.neuroimage.2009.06.060.

16. Zhang Y, Brady M, Smith S. Segmentation of brain MR images through a hidden Markov random field model and the expectation-maximization algorithm. IEEE Trans Med Imaging. 2001 Jan;20(1):45–57. doi:10.1109/42.906424.

17. Behzadi Y, Restom K, Liau J, Liu TT. A component based noise correction method (CompCor) for BOLD and perfusion based fMRI. Neuroimage. 2007 Aug 1;37(1):90–101. doi:10.1016/j.neuroimage.2007.04.042.

18. Power JD, Mitra A, Laumann TO, Snyder AZ, Schlaggar BL, Petersen SE. Methods to detect, characterize, and remove motion artifact in resting state fMRI. Neuroimage. 2013 Aug 29;84:320–41. doi:10.1016/j.neuroimage.2013.08.048.

19. Pruim RHR, Mennes M, van Rooij D, Llera A, Buitelaar JK, Beckmann CF. ICA-AROMA: A robust ICA-based strategy for removing motion artifacts from fMRI data. Neuroimage. 2015 May 15;112:267–77. doi:10.1016/j.neuroimage.2015.02.064.

20. Klein A, Ghosh SS, Bao FS, Giard J, Häme Y, Stavsky E, et al. Mindboggling morphometry of human brains. PLoS Comput Biol 13(2): e1005350. 2017. doi:10.1371/journal.pcbi.1005350.

21. Abraham A, Pedregosa F, Eickenberg M, Gervais P, Mueller A, Kossaifi J, Gramfort A, Thirion B, Varoquaux G. Machine learning for neuroimaging with scikit-learn. Front in Neuroinf 8:14. 2014. doi:10.3389/fninf.2014.00014.

***2.2.2 fMRI task design***

To investigate whether our MID-design evoked similar brain activation patterns as previous MID tasks, we first compared the second level contrast *Win vs Baseline* anticipation and outcome activation to previously published results. For follow-up analysis we specified additional contrasts for *High win vs Baseline*, *Low win vs Baseline* and *High win vs Low win* separately, to be able to compare activation during different reward levels. Further, each reward level during anticipation and outcome was modelled against an intrinsic baseline, and mean parameter estimates were extracted for the pre-defined ROIs. These values could then be compared over the different reward levels through multiple regression models in R version 3.1.3 (R Core Team 2015).

**3. Results**

**3.1 Behavioral results – *High win* and *Low win* separately**

**Main effects:** The Linear Mixed Effects model showed a significant overall effect of reward level on *RT* (F(2,1935)=39.12, p<.001). Post-hoc tests revealed a faster *RT* for *High win vs Baseline* (beta=-0.04, t(1935)=-8.03, p<.001), *Low win vs Baseline* (beta=-0.01, t(1935)=-2.57, p<.05) and *High win vs Low win* (beta=-0.03, t(1935)=-6.69, p<.001) (Supplementary Figure 2 and Supplementary Table 2).

**Correlations:** There were no significant correlations between *Inattention* or *Emotion Instability* and mean *RT* or *RTV* for any of the reward levels. There no significant difference of mean *RT* between males and females (p=.53).

There was a trend significant moderate correlation between *High RTS* and *Emotion Instability* (r=.33, p= .08), and when controlling for *Inattention* (standardized beta-weight=.33, p=.10). There was no significant correlation between *Low RTS* and *Emotion Instability*.

**3.2 Behavioral results – Age**

There was a positive correlation between *RT* and age (r=.52, p<.01), but no significant difference of *RT* between males and females (t(27)=0.64, p=.53).

Age correlated with *RTS* on a trend level (r=.36, p=.053), and when introducing age as covariate, *Emotion Instability* was not correlated with *RTS* (standardized beta-weight=.27, p=.17), even though *Emotion Instability* and age did not show a significant correlation (r=.22, p=.26).

**3.3 fMRI results, main activations**

**Reward anticipation:** A similar activation pattern as observed for the contrast *Win vs Baseline* (Main Figure 3b) was present for the contrast *High win vs Baseline* as well as for *Low win vs Baseline* (Supplementary Figure 3a and Supplementary Figure 3b). Moreover, there was a significantly larger VS activation for anticipating *High win vs Low win* (Supplementary Figure 3c). Extracting the mean parameter estimates of activation from the pre-defined bilateral VS ROI during reward anticipation showed an incremental BOLD response for increased reward from *Baseline* to *Low win* to *High win* in VS (Supplementary Figure 3d).

**Reward outcome:** Similar activation patterns as for *Win vs Baseline* (Main Figure 4) were observed for *High win vs Baseline*, *Low win vs Baseline* and *High win vs Low win* (Supplementary Figure 4 and Supplementary Figure 5).

**“Failed” reward outcome:** A similar activation pattern as for *“Failed” win vs Baseline* could be seen for *“Failed” high win vs Baseline* only trials and *“Failed” low win vs Baseline* only trials (Supplementary Figure 6).

**3.4 fMRI results – Correlations with *Emotion Instability*/*Inattention***

**3.4.1 ROI analysis: *High win vs Baseline*, *Low win vs Baseline*, *High win vs Low win***

There were no correlations of *Emotion Instability* or *Inattention* in any of the pre-defined ROIs for anticipation or outcome of *High win vs Baseline*, *Low win vs Baseline* or *High win vs Low win* when applying small volume correction within the pre-defined ROIs. Clusters were considered significant if the FWE corrected peak p-value was <.05.

**3.4.2 Whole brain analysis**

There were no additional significant clusters > 20 voxels (FWE correction, p<.05) when correlating *Emotion Instability* or *Inattention* with either activation during anticipation of reward or outcome of reward for *Win vs Baseline*, *High win vs Baseline*, *Low win vs Baseline* or *High win vs Low win*.

**Supplementary figures**


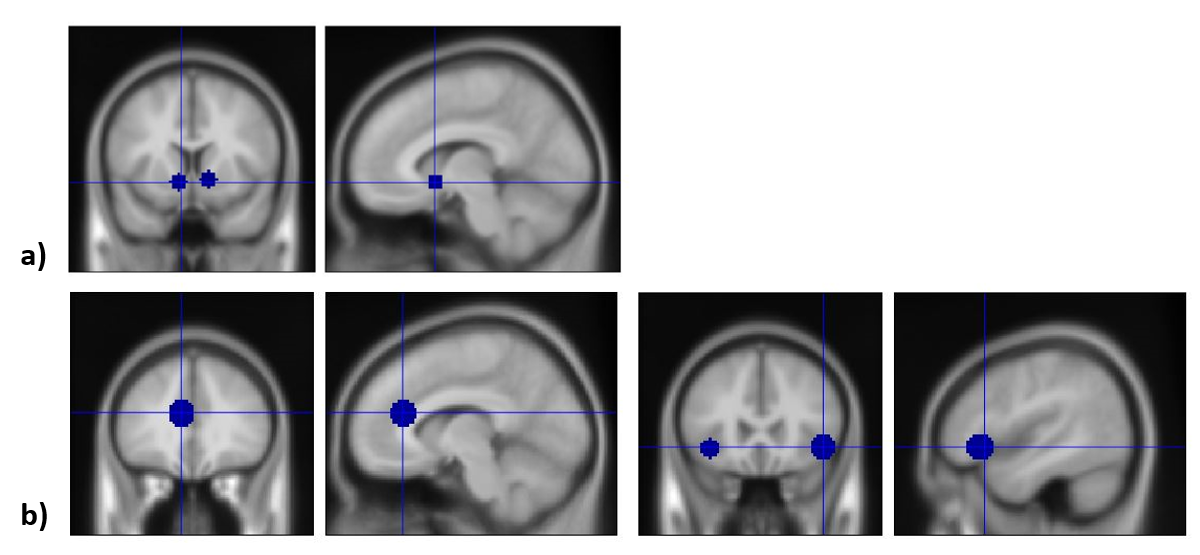


**Supplementary Figure 1 a)** Bilateral VS ROI, based on peak coordinates of reward *anticipation* in Oldham review (exact coordinates in MNI space: 12, 10, -4 and -10, 10, -6 (Oldham et al. 2018), each sphere with radius= 6 mm). **b)** rACC and bilateral insula ROI, based on peak coordinate of activation in reward *outcome* phase from Dillon study (exact MNI coordinates: (-8, 33, 19), (46, 27, -6) and (-38, 17, -7)(Dillon et al. 2010), sphere with radius 10 mm).


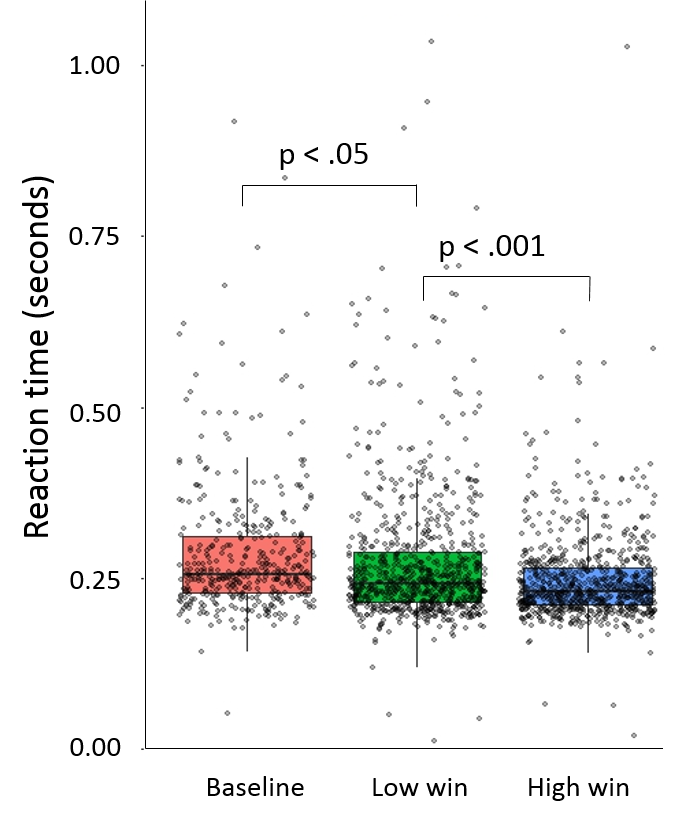


**Supplementary Figure 2** Reaction time across the different reward levels (*Baseline*, *Low win* and *High win*).

**
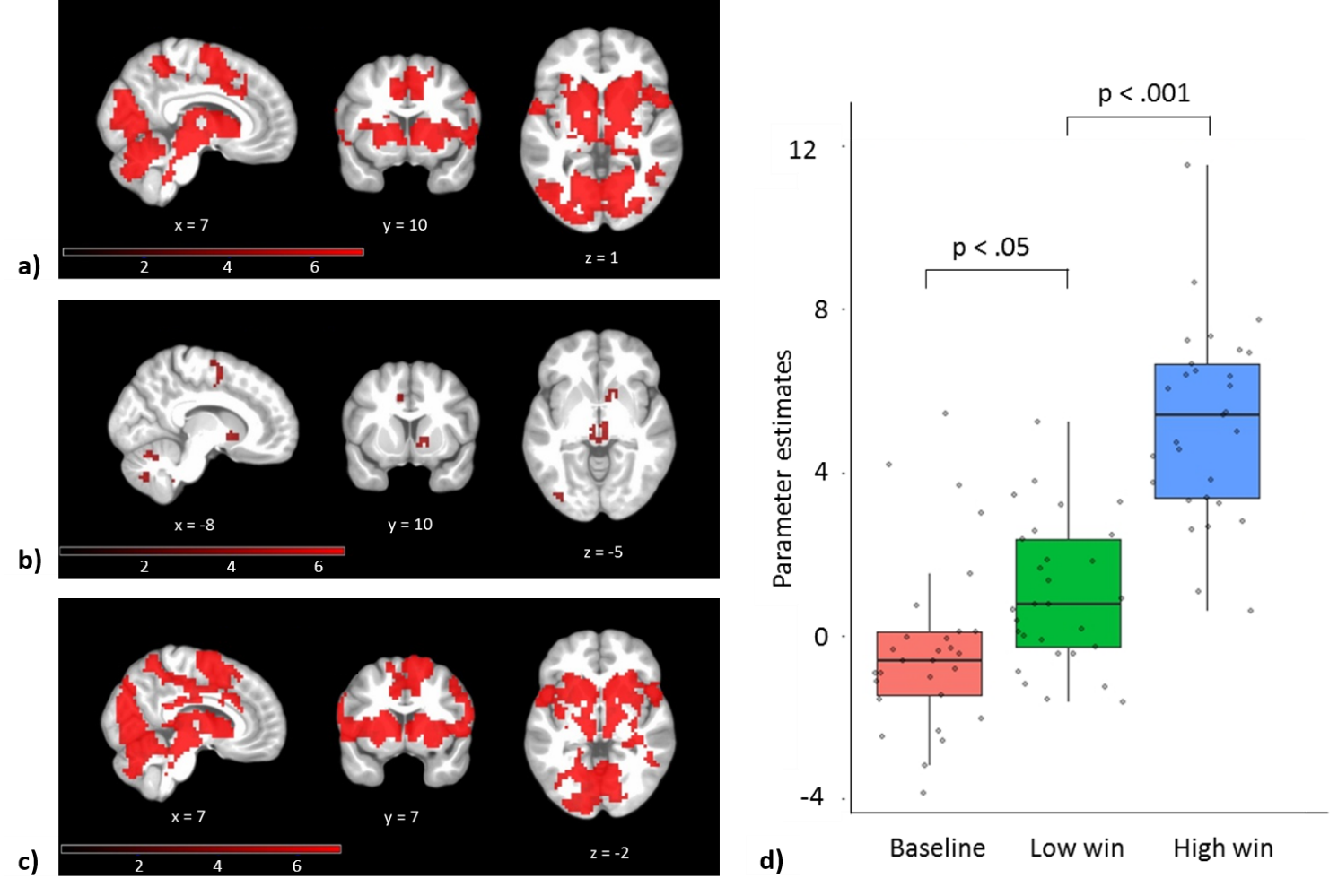
**

**Supplementary Figure 3** Anticipation of reward. MNI coordinates (x, y, z) reported. Activations are overlaid on an average T1 image based on our 29 participants. Color bars indicate T-scores. All brain images are in neurologic orientation. **a)** Anticipation *High win vs Baseline*, FWE, p<.05, clusters > 20 voxels. **b)** Anticipation *Low win vs Baseline*, uncorrected, p<.001, clusters > 20 voxels. **c)** Anticipation *High win vs Low win*, FWE, p<.05, clusters > 20 voxels. **d)** Mean parameter estimates from bilateral ventral striatum for the different reward levels. The overall ANOVA was significant (F(2,84)= 54.54, p<.001)


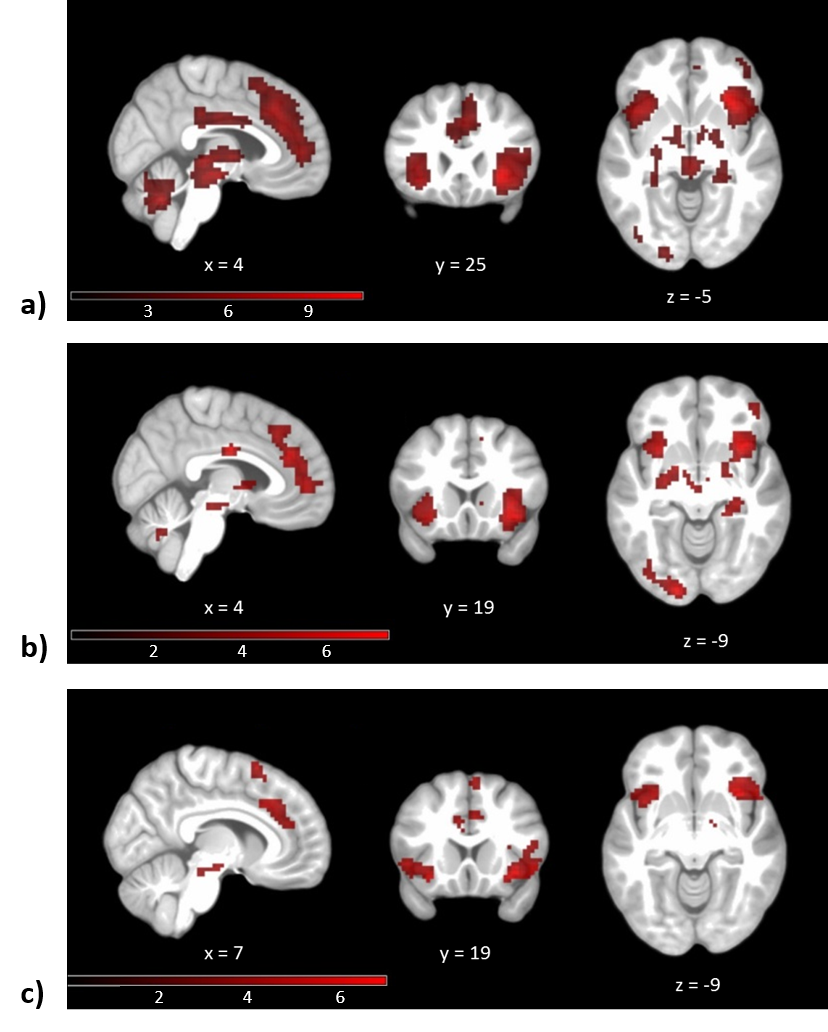


**Supplementary Figure 4** Outcome of reward. Uncorrected, p<.001, clusters > 20 voxels. MNI coordinates (x, y, z) reported. Activations are overlaid on an average T1 image based on our 29 participants. Color bars indicate T-scores. Brain images are in neurologic orientation. a) Outcome of *High win vs Baseline*. b) Outcome of *Low win vs Baseline*. c) Outcome of *High win vs Low win*.


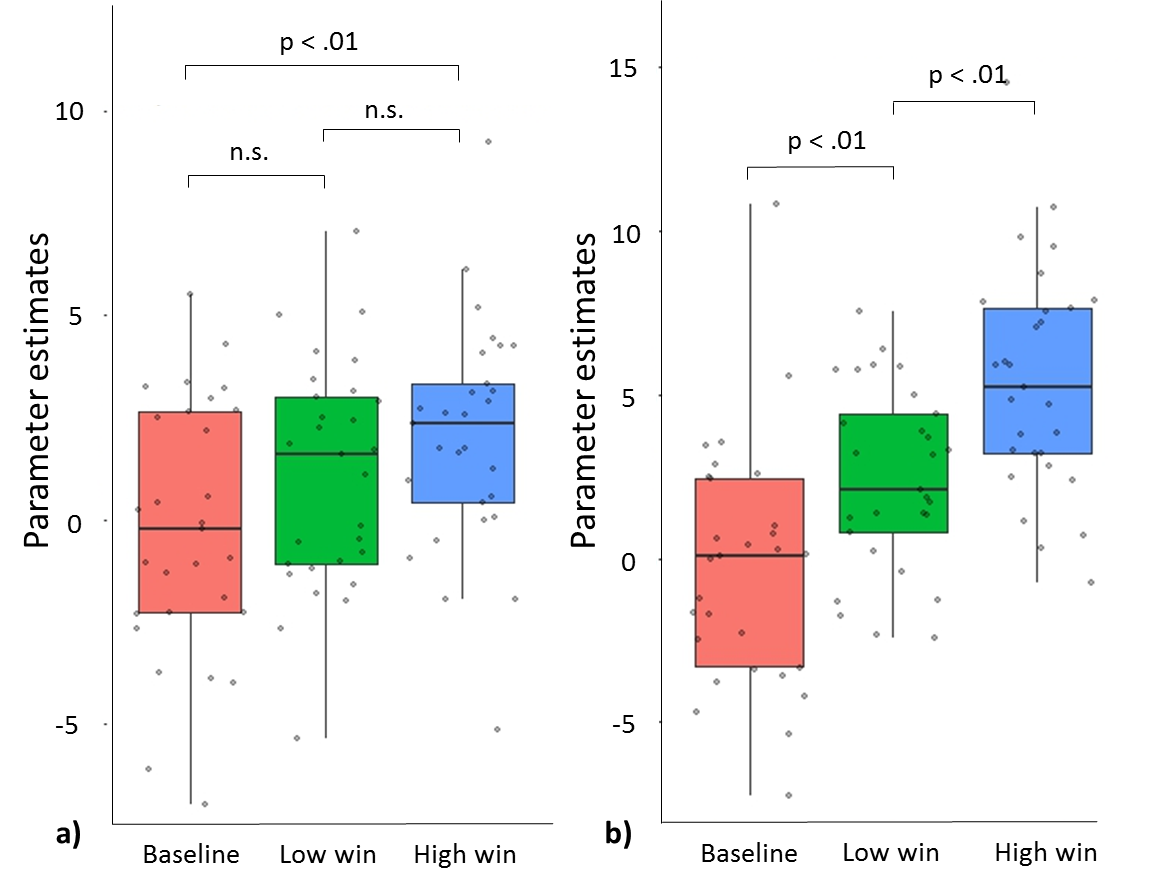


**Supplementary Figure 5** Mean parameter estimates of reward outcome phase for the different reward levels. **a)** ROI specified from peak coordinate in rACC cluster in Dillon study (Dillon et al. 2010), with radius=10 mm. The overall ANOVA was significant (F(2,84)=4.36, p<.05). **b)** ROI specified from peak coordinate in bilateral insula clusters in Dillon study (Dillon et al. 2010), with radius=10 mm. The overall ANOVA was significant (F(2,84)=21.05, p<.001). n.s., not statistically significant, p > .05.


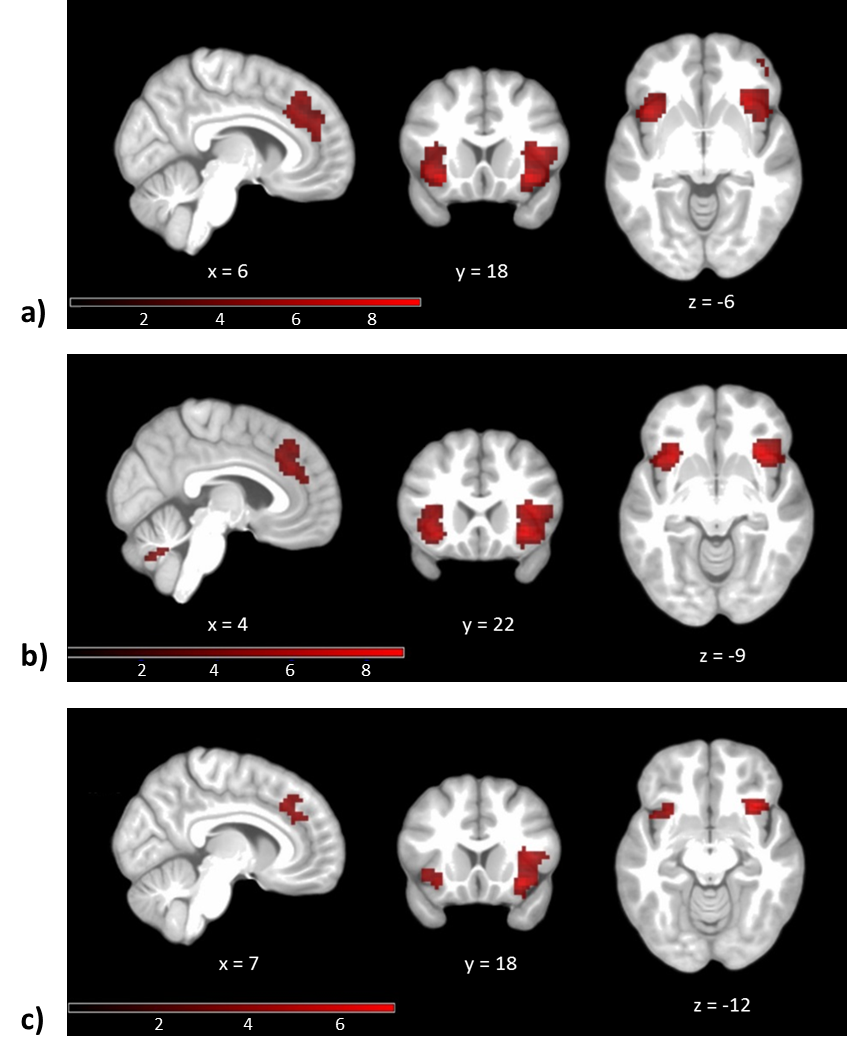


**Supplementary Figure 6** *“Failed” win vs Baseline*. Uncorrected, p<.001, clusters > 20 voxels. MNI coordinates (x, y, z) reported. Activations are overlaid on an average T1 image based on our 29 participants. Exact location of activations may be found in Supplementary Table 5. Color bars indicate T-scores. Brain images are in neurologic orientation. **a)** Main activation when failing to receive a reward *(“Failed” low win* and *“Failed” high win* combined) vs *Baseline*. **b)** *“Failed” high win vs Baseline* (Anticipating *High win*, but no win received, vs *Baseline*) **c)** *“Failed” low win vs Baseline* (Anticipating *Low win*, but no win received, vs *Baseline*)


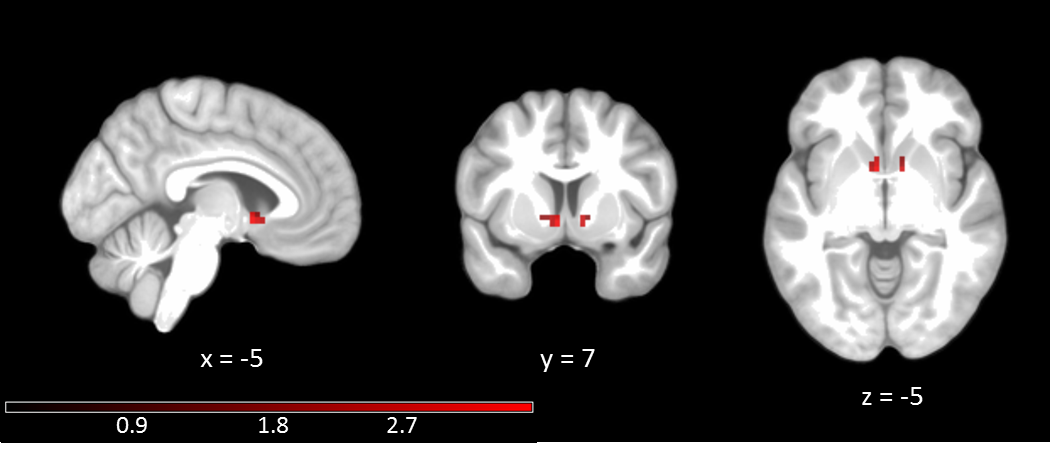


**Supplementary Figure 7** Main effect of sex on activation during anticipation of *Win vs Baseline* in predefined bilateral VS ROI, controlling for *Inattention* and *Emotion Instability* in the whole sample. Small volume corrected, Left VS: peak-level FWE-corrected p=.02, Z=3.19, cluster size: 10 voxels, MNI coordinates: -5, 7, -5, Right VS: peak-level FWE-corrected p=.12, Z=2.62, cluster size: 6 voxels, MNI coordinates: 10, 7, -5. Displayed at uncorrected level, p<.05. Masked to only show correlations within pre-defined bilateral VS ROI. Color bar indicates T-scores. Brain images are in neurologic orientation.


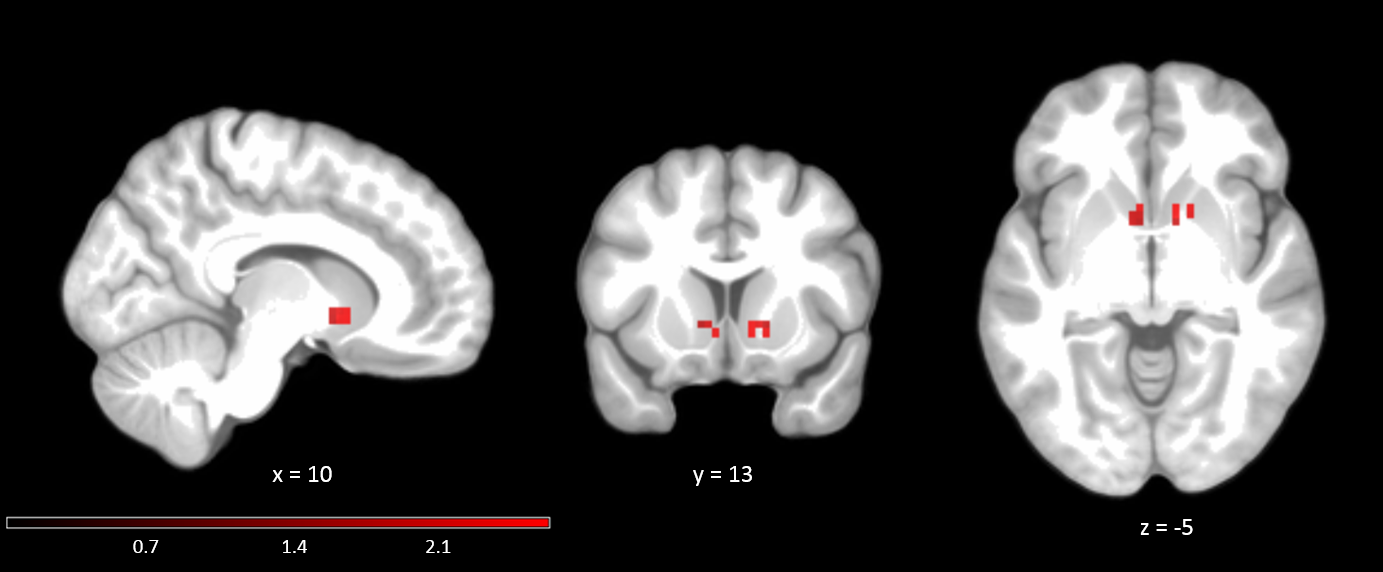


**Supplementary Figure 8** Interaction effect of sex and *Emotion Instability* on activation during anticipation of Win vs Baseline on a voxel level within predefined bilateral VS ROI, controlling for sex and *Inattention* in the whole sample*.* Small volume corrected, Left VS: peak-level FWE-corrected p=.20, Z=2.37, cluster size: 13 voxels, MNI coordinates: -5, 13, -5, Right VS: peak-level FWE-corrected p=.16, Z=2.47, cluster size: 13 voxels, MNI coordinates: 10, 13, -5. Displayed at uncorrected level, p<.05. Masked to only show correlations within pre-defined bilateral VS ROI. Color bar indicates T-scores. Brain images are in neurologic orientation.

**Supplementary tables**

**Supplementary Table 1** Description of sample characteristics.

|  | **Number of participants** | **Age, mean (years)** | **B-ADD *Inattention*, mean** | **B-ADD *Emotion Instability*, mean** | **B-ADD total, mean** |
| --- | --- | --- | --- | --- | --- |
| **Whole sample** | 29 | 28.94 (SD=6.47, min=18.70, max=46.50) | 8.59 (SD=5.03, min: 0, max: 19) | 4.59 (SD=2.37, min= 1, max=9) | 31.69 (SD=13.05, min=4, max=60) |
| **Females** | 15 | 28.29 (SD=7.17, min=18.70, max=46.50) | 9.13 (SD=4.61, min=0, max=16) | 5.00 (SD=2.42, min=1, max=9) | 32.13 (SD=11.27, min=4, max=49) |
| **Males** | 14 | 29.64 (SD=5.81, min=22.40, max=43.50) | 8.00 (SD=5.56, min=1, max=19) | 4.14 (SD=2.32, min=1, max=9) | 31.21 (SD= 15.16, min=8, max=60) |

Two Sample t-tests revealed no statistically significant differences between males and females (age: t(27)=-0.56, p=.58, B-ADD *Inattention*: t(27)=0.60, p=.55, B-ADD *Emotion Instability*: t(27)=0.97, p=.34, B-ADD total score: t(27)=0.19, p=.85)Abbreviations: B-ADD: Brown Attention-Deficit Disorder Scales. SD: standard deviation.

**Supplementary Table 2** Mean reaction time (*RT*) and standard deviation (SD) for the different reward levels.

| ***Baseline* *RT*, mean(SD)** | ***Low win* *RT*, mean(SD)** | ***High win* *RT*, mean(SD)** | **All *RT*, mean(SD)** |
| --- | --- | --- | --- |
| 0.29(0.10), n=395 | 0.27(0.10), n=794 | 0.25(0.07), n=777 | **0.27(0.09), n=1966** |

“n” depicts the number of trials across all subjects. Bold values refer to overall mean RT, including *Baseline*, *Low win* and *High win* trials presented separately in the first three columns.

**Supplementary Table 3** Significant clusters in contrast anticipation *Win vs Baseline*

| **Localization** | **MNI** | | | **Peak z-score** | **Cluster size (voxels)** | **Peak p-value, FWE corrected** |
| --- | --- | --- | --- | --- | --- | --- |
|  | **X** | **Y** | **Z** |  |  |  |
| **L caudate/NAcc*** | **-8** | **10** | **-2** | **6.10** | **15** | **<.001*** |
| **R caudate/NAcc*** | **10** | **13** | **-2** | **6.08** | **14** | **<.001*** |
| **L middle cingulate gyrus** | **-11** | **7** | **37** | **6.71** | **755** | **<.001** |
| R superior frontal gyrus | 16 | -2 | 61 | 6.08 |  | <.001 |
| L precentral gyrus | -35 | -20 | 70 | 5.96 |  | <.001 |
| R supplementary motor cortex | 13 | 1 | 54 | 5.93 |  | <.001 |
| R supplementary motor cortex | 1 | -2 | 57 | 5.66 |  | <.001 |
| R precentral gyrus | 28 | -5 | 54 | 5.66 |  | <.001 |
| R superior frontal gyrus | 22 | 1 | 54 | 5.64 |  | <.001 |
| R supplementary motor cortex | 7 | -5 | 67 | 5.63 |  | <.001 |
| L precentral gyrus | -23 | -14 | 70 | 5.58 |  | .001 |
| R supplementary motor cortex | 7 | 1 | 64 | 5.55 |  | .001 |
| L superior frontal gyrus | -17 | -8 | 74 | 5.45 |  | .001 |
| R superior frontal gyrus | 22 | 1 | 67 | 5.43 |  | .001 |
| L postcentral gyrus | -42 | -17 | 47 | 5.41 |  | .002 |
| L precentral gyrus | -45 | -11 | 64 | 5.34 |  | .002 |
| L superior frontal gyrus | -23 | -11 | 57 | 5.32 |  | .003 |
| **R cerebellum white matter** | **22** | **-59** | **-29** | **6.68** | **2284** | **<.001** |
| L occipital fusiform gyrus | -35 | -71 | -12 | 6.58 |  | <.001 |
| R fusiform gyrus | 31 | -59 | -19 | 6.55 |  | <.001 |
| L superior occipital gyrus | -23 | -75 | 31 | 6.53 |  | <.001 |
| R cerebellum exterior | 10 | -62 | -25 | 6.45 |  | <.001 |
| Cerebellar Vermal lobules VIII-X | 4 | -68 | -35 | 6.44 |  | <.001 |
| R cerebellum Exterior | 28 | -50 | -29 | 6.36 |  | <.001 |
| R cerebellum Exterior | 10 | -68 | -22 | 6.34 |  | <.001 |
| Cerebellar Vermal Lobules VIII-X | -2 | -56 | -35 | 6.31 |  | <.001 |
| L cerebellum Exterior | -11 | -78 | -22 | 6.30 |  | <.001 |
| R cerebellum exterior | 16 | -53 | -48 | 6.29 |  | <.001 |
| L calcarine cortex | -8 | -81 | 14 | 6.27 |  | <.001 |
| Brain stem | -2 | -38 | -48 | 6.26 |  | <.001 |
| R calcarine cortex | 10 | -65 | 8 | 6.26 |  | <.001 |
| R calcarine cortex | 16 | -75 | 14 | 6.25 |  | <.001 |
| L cerebellum exterior | -17 | -59 | -48 | 6.23 |  | <.001 |
| **L thalamus** | **-8** | **-23** | **-2** | **6.44** | **883** | **<.001** |
| Brain stem/R ventral DC | 7 | -17 | -9 | 6.36 |  | <.001 |
| Brain stem/R ventral DC | 7 | -26 | -5 | 6.20 |  | <.001 |
| Brain stem/L ventral DC | -8 | -14 | -12 | 6.17 |  | <.001 |
| R caudate | 10 | 10 | 1 | 6.16 |  | <.001 |
| R caudate | 10 | 16 | -2 | 6.15 |  | <.001 |
| L caudate | -8 | 10 | -2 | 6.10 |  | <.001 |
| R caudate | 13 | 4 | 8 | 6.02 |  | <.001 |
| R pallidum | 19 | -5 | 1 | 6.00 |  | <.001 |
| R frontal operculum | 37 | 28 | 11 | 5.85 |  | <.001 |
| Brain stem/L ventral DC | -11 | -8 | -9 | 5.83 |  | <.001 |
| Brain stem/R ventral DC | 7 | -8 | -12 | 5.68 |  | <.001 |
| R thalamus | 16 | -14 | 1 | 5.67 |  | <.001 |
| R putamen | 22 | 13 | 1 | 5.67 |  | <.001 |
| L putamen/pallidum | -17 | 1 | -9 | 5.56 |  | .001 |
| Brain stem | 4 | -32 | -19 | 5.53 |  | .001 |
| **L superior parietal lobule** | **-29** | **-53** | **70** | **5.73** | **60** | **<.001** |
| L superior parietal lobule | -23 | -56 | 57 | 5.73 |  | <.001 |
| **L precuneus** | **-11** | **-50** | **54** | **5.57** | **72** | **.001** |
| L precentral gyrus medial segment | -17 | -35 | 47 | 5.45 |  | .001 |
| **L central operculum** | **-57** | **1** | **1** | **5.57** | **22** | **.001** |
| **L postcentral gyrus** | **-42** | **-35** | **51** | **5.36** | **43** | **.002** |
| **R middle occipital gyrus** | **31** | **-75** | **31** | **5.32** | **22** | **.003** |
| R middle occipital gyrus | 40 | -75 | 24 | 5.12 |  | .008 |
| **R precentral gyrus medial segment** | **16** | **-29** | **44** | **5.16** | **14** | **.006** |

Coordinates (x, y, z) correspond to the anatomical space as defined in the MNI standard brain atlas. Localization of activations is reported using the Neuromorphometrics labelling supplied by SPM. Nine main clusters are given (bold font) and the regions they expand to are listed. Clusters were expanded when possible and the top 16 sub-peak activations within each cluster were considered. Only statistically significant peaks in grey matter are reported. Clusters > 10 voxels are reported. * SVC within bilateral VS ROI derived from peak coordinates in a meta-analysis by Oldham et al. (Oldham et al. 2018), cluster size in number of voxels at uncorrected level p<.05. Other activation clusters reported are full brain corrected (FWE) and considered significant at peak-level (FWE-corrected) p<.05. Voxel size 3 x 3 x 3 mm^3^.

**Supplementary table 4** Significant clusters in contrast outcome *Win vs Baseline*.

| **Localization** | **MNI** | | | **Peak z-score** | **Cluster size (voxels)** | **Peak p-value, FWE corrected** |
| --- | --- | --- | --- | --- | --- | --- |
|  | **X** | **Y** | **Z** |  |  |  |
| **R orbital part of the inferior frontal gyrus/R anterior insula** | **37** | **22** | **-9** | **6.23** | **107** | **<.001*** |
| **L anterior insula** | **-32** | **16** | **-9** | **5.93** | **136** | **<.001*** |
| **R anterior cingulate gyrus** | **1** | **34** | **24** | **4.81** | **108** | **<.001**** |
| L anterior cingulate gyrus | -11 | 40 | 18 | 4.69 |  | <.001** |
| L anterior cingulate gyrus | -5 | 31 | 28 | 4.48 |  | <.001** |
| **R anterior insula** | **31** | **25** | **-2** | **6.62** | **166** | **<.001** |
| **R superior frontal gyrus medial segment** | **13** | **34** | **24** | **6.09** | **70** | **<.001** |
| R superior frontal gyrus medial segment | 1 | 31 | 34 | 5.21 |  | .005 |
| R superior frontal gyrus medial segment | 4 | 28 | 37 | 5.10 |  | .009 |
| R superior frontal gyrus medial segment | 7 | 28 | 44 | 5.06 |  | .011 |
| R superior frontal gyrus medial segment | 13 | 40 | 14 | 5.05 |  | .011 |
| **L anterior insula** | **-32** | **16** | **-9** | **5.93** | **60** | **<.001** |
| **R superior frontal gyrus medial segment** | **4** | **50** | **1** | **5.32** | **11** | **.003** |
| **Brain stem/R ventral DC** | **4** | **-23** | **-12** | **5.30** | **12** | **.003** |

Coordinates (x, y, z) correspond to the anatomical space as defined in the MNI standard brain atlas. Localization of activations is reported using the Neuromorphometrics labelling supplied by SPM. Clusters were expanded when possible and the top 16 sub-peak activations within each cluster were considered. Only statistically significant peaks in grey matter are reported. Clusters > 10 voxels are reported. * SVC within bilateral insula ROI derived from peak coordinates in a study by Dillon et al. (Dillon et al. 2010), cluster size in number of voxels at uncorrected level p<.05. ** SVC within rACC ROI derived from peak coordinates in a study by Dillon et al. (Dillon et al. 2010), cluster size in number of voxels at uncorrected level p<.05. Other activation clusters reported are full brain corrected (FWE) and considered significant at peak-level (FWE-corrected) p<.05. Voxel size 3 x 3 x 3 mm^3^. Top peak activation in each cluster given in bold font.

**Supplementary table 5** Significant clusters in contrast outcome *“Failed” win vs Baseline*.

| **Localization** | **MNI** | | | **Peak z-score** | **Cluster size (voxels)** | **Peak p-value, FWE corrected** |
| --- | --- | --- | --- | --- | --- | --- |
|  | **X** | **Y** | **Z** |  |  |  |
| **R anterior insula** | **34** | **22** | **-9** | **6.20** | **83** | **<.001** |
| R frontal operculum | 37 | 22 | 8 | 5.35 |  | .002 |
| **L anterior insula** | **-32** | **19** | **-9** | **5.99** | **38** | **<.001** |

Coordinates (x, y, z) correspond to the anatomical space as defined in the MNI standard brain atlas. Localization of activations is reported using the Neuromorphometrics labelling supplied by SPM. Clusters were expanded when possible and the top 16 sub-peak activations within each cluster were considered. Only statistically significant peaks in grey matter are reported. Clusters > 10 voxels are reported. Activation clusters reported are full brain corrected (FWE) and considered significant at peak-level (FWE-corrected) p<.05. Voxel size 3 x 3 x 3 mm^3^. Top peak activation in each cluster given in bold font.
